# Prediction Model of the Quality of Life for Patients with Pancreatic Cancer

**DOI:** 10.3390/healthcare10101973

**Published:** 2022-10-09

**Authors:** Jisun Lee

**Affiliations:** Department of Nursing, Honam University, Gwangju 62399, Korea; 2018091@honam.ac.kr

**Keywords:** pancreatic neoplasms, quality of life, social support, symptom, perceived health status

## Abstract

This study attempted to establish a predictive model that systematically and comprehensively predicts the quality of life (QoL) of patients with pancreatic cancer. The study used a descriptive cross-sectional design. A total of 248 patients was included who were selected via the convenience sampling method. A structured questionnaire was used and the collected data were analyzed for fitness, using the SPSS WIN 25.0 program and AMOS 24.0. The perceived health status of the patients with pancreatic cancer would directly affect their QoL and indirectly influence the QoL through social support, symptoms, functional status, and age. The application of social support intervention programs to relieve specific symptoms and improve the functional status according to the conditions of patients may contribute to improving the QoL of pancreatic cancer patients. This predictive model could be used as the basis for developing a nursing intervention plan and writing evaluation guidelines for practitioners who provide nursing care for patients with pancreatic cancer.

## 1. Introduction

Despite breakthroughs in modern medicine, pancreatic cancer remains a malignant tumor with high mortality; the 1 year and 5-year survival rates are 23% and 7.8%, respectively [1]. To improve the survival rate of patients with pancreatic cancer, combination therapies including surgery, chemotherapy, and radiotherapy have been developed; however, the quality of life (QoL) of patients with pancreatic cancer remains poor [2]. Identifying the factors that affect the QoL of pancreatic cancer patients with low survival rates is therefore important.

Most previous studies focused on the effects of health, illness, and treatment in a concept called “health-related quality of life” (HRQoL), which does not consider aspects of life that are not directly related to the disease such as cultural, political, or social characteristics [3]. However, because various areas of life affect health, a causal relation model is needed to integrate the elements of HRQoL and its determinants [4].

Furthermore, studies investigating pancreatic cancer HRQoL have addressed different issues, including the effects of symptoms such as fatigue, pain, dyspnea, and anorexia after surgery on QoL; how the absorption of nutrients, weight loss, diarrhea, and diabetes after surgery decrease QoL; the effect of a high number of symptoms on lowering QoL; and the association between depression and anxiety in pancreatic cancer patients and overall low QoL and poor cognitive, social, and physical functioning [5,6]. However, most studies on pancreatic cancer have focused on disease-related physical symptoms, whereas few studies have examined the causal relationships between the variables that affect the QoL of pancreatic cancer patients [7,8].

Bakas et al. [9] examined the most commonly used HRQoL models and conducted a systematic literature review. These authors identified the model of Ferrans et al. [3,10] as a potentially useful model for conducting HRQoL research. Ferrans et al. [3] used the QoL study model to demonstrate that individuals who face unexpected health problems retain the ability to make value judgments on various important aspects of their lives. In addition, they reported that nursing intervention can be designed effectively by defining the relationship between variables that affect QoL from a nursing perspective. The HRQoL model of Ferrans et al. [3] examines the direct or indirect effects of biological functions, symptoms, functional status, and general health perception on HRQoL through individual and environmental characteristics, thus investigating QoL from a dynamic and pluralistic aspect. 

Lee [11] categorized the HRQoL of cancer patients according to the theoretical framework of the HRQoL model of Ferrans et al. [3], and confirmed the relationships between key words. The analysis revealed the importance of biological functional and symptom areas for breast cancer, symptoms and functional conditions for prostate cancer, biological functions, symptoms and functional conditions for cervical cancer, and biological functional areas for colon cancer and stomach cancer [11]. These studies are limited in that, according to the nature of the disease, they only establish the relationship or effects between certain variables by utilizing some of the concepts mentioned in the model. The results of these studies cannot accurately identify the various characteristics of the subjects, and their value for helping the subjects to solve their health problems is thus limited [12]. To solve the multifaceted and multidimensional life problems faced by pancreatic cancer patients, it is necessary to investigate the relationships and effects of specific factors by considering different aspects that affect the QoL of patients.

This study attempted to establish a predictive model that systematically and comprehensively predicts the QoL of patients with pancreatic cancer. The study could be used as basic data to design specific and practical nursing plans for improving the QoL of pancreatic cancer patients in the future.

## 2. Materials and Methods

### 2.1. Research Design

This study used a descriptive, cross-sectional survey design.

### 2.2. Study Participants

The study included 250 patients who were diagnosed with pancreatic cancer using the convenience sampling method and who visited a hospital as outpatients to receive palliative care intervention. The selection criteria were as follows: (1) adults >20 years old; (2) diagnosed with pancreatic cancer; (3) patients capable of understanding the contents of the questionnaire and responding directly to the questionnaire; and (4) patients who understood the purpose of the study and agreed to participate in the study in writing. The exclusion criteria were as follows: (1) non-protopathic pancreatic cancer; and (2) serious comorbid diseases requiring treatment other than pancreatic cancer. The standard population size required for the maximum likelihood in a structural equation model is >200 specimens [13]. Thus, in this study, data on 250 patients were collected considering the ideal recommended size and the dropout rate due to the omission of some items in the questionnaire. A total of 250 persons were contacted, and two of them were rejected during the questionnaire, resulting in a total of 248 subjects being included.

### 2.3. Research Instruments

All measurements were used after gaining the developer’s approval.

#### 2.3.1. General Characteristic

The general characteristic of the subjects was investigated with variables such as gender, age, education level, marital status, primary caregiver and duration after diagnosis. 

#### 2.3.2. Social Support

The Multi-dimensional Scale of Perceived Social Support (MSPSS), developed by Zimet et al. [14], was used. This tool is categorized as family, friends, and meaningful others, and is comprised of a total of 12 questions, with four questions for each area. It uses Likert-type 5-point scales, and the scores for each question are from the lowest 4 to the highest 20, with higher scores indicating a higher degree of social support. Cronbach’s α was 0.99 at the time of development, and 0.94 in this study.

#### 2.3.3. Symptoms

Physical symptoms were measure as a symptom scale item among the European Organization for Research and Treatment of Cancer (EORTC) QLQ-C30 version 3.0 [15]. This tool consists of a total of 13 items and is based on a 4-point Likert scale. According to the scoring guidelines of the tool, it is converted to 0–100 points. A higher score means more severe symptoms. Cronbach’s α was 0.85 at the time of development, and 0.90 in this study. For anxiety and depression, The Hospital Anxiety and Depression Scale (HADS) developed by Zigmond & Snaith [16] was used. The HADS consists of 14 questions. Of these, the odd-numbered seven items are questions about anxiety (HADS-A), and the even-numbered seven items are questions about depression (HADS-D). It uses a 4-point Likert scale. The scores range from 0 to 21, with higher scores indicating higher anxiety or depression. Cronbach’s α was 0.89 for anxiety and 0.86 for depression at the time of development, and in this study, it was 0.90 for anxiety and 0.96 for depression.

#### 2.3.4. Functional Status 

Functional status was measure as a functional scale item among the EORTC QLQ-C30 version 3.0 [15]. This tool is categorized as physical functioning, role functioning, emotional functioning cognitive functioning and social functioning, which is comprised of a total of 15 questions. It uses a 4-point Likert scale. According to the scoring guidelines of the tool, it is converted to 0–100 points. A higher score means a better functional status. Cronbach’s α was 0.90 at the time of development, and 0.90 in this study. 

#### 2.3.5. Perceived Health Status

Perceived health status was measured with the general health perception item among the Short Form (SF-36) items [17]. This tool consists of a total of five items and is based on a 5-point Likert scale. According to the scoring guidelines of the tool, it is converted to 0–100 points. A higher score means a better perceived health status. Cronbach’s α was 0.78 at the time of development, and 0.97 in this study. 

#### 2.3.6. Quality of Life

The QoL Index-generic version III developed by Ferrans & Powers was used [18]. The tool is divided into two parts: the first part consists of 33 questions addressing the QoL of the subject in terms of satisfaction with life, and the second part consists of 33 questions addressing the degree of satisfaction with the QoL in the same questions. This tool is categorized as health functional, socioeconomic, psychological-spiritual and family, and is comprised of a total of 66 questions. It uses a 6-point Likert scale. According to the scoring guidelines of the tool, it is converted to 0–30 points. A higher score means a better perceived QoL. Cronbach’s α was 0.89 at the time of development and 0.92 in this study.

### 2.4. Data Collection 

Data were collected from March to November 2017. The researcher examined the list of outpatients in advance and performed convenience sampling of the subjects that met the selection criteria among patients diagnosed with pancreatic cancer who visited the outpatient clinic. After directly describing the purpose and procedure of the study verbally and in writing to the subjects who were waiting for treatment, the researcher enrolled those who agreed to participate in the study. The researcher then carried out the survey, and the subjects directly filled in the questionnaire. The survey was conducted in the outpatient waiting room, and it took about 15 min to complete the questionnaire. When the questionnaire was completed, the researcher checked the questionnaire and attempted to decrease the number of dropouts by making a list of missing questions on the spot. 

### 2.5. Data Analyses

The data collected in this study were analyzed using the SPSS WIN 25.0 program and AMOS 24.0. 

In the main variables of the subjects, the frequency, mean, standard deviation, and percentage were utilized. 

The sample normality was tested with mean, standard deviation, skewness, kurtosis, tolerance, and variance inflation factor (VIF).

The covariance structure analysis was used to calculate the direct and indirect path coefficients between the factors affecting the QoL, and the maximum likelihood, which assumes multivariate normality, was also used. 

The goodness-of-fit tests used to evaluate the suitability of the study model were as follows: χ^2^, χ^2^/df, goodness-of-fit index (GFI), adjusted goodness-of-fit index (AGFI), comparative fit index (CFI), root mean squared error of approximation (RMSEA), parsimonious goodness-of-fit index (PGFI), and parsimonious normed fit index (PNFI). 

To test the path significance of the model, the estimated path coefficient and the Critical Ratio (CR) values were determined. 

### 2.6. Ethical Consideration

The study was conducted according to the guidelines of the Declaration of Helsinki, and approved by the Institutional Review Board (IRB No. 2017-0325). Informed consent was obtained from the subjects before proceeding with the study. The study participation agreement included the anonymity and confidentiality of the subjects. The subjects were informed that they could stop answering the questionnaire during the survey and withdraw at any time. To prevent the leakage of personal information and questionnaire contents, the questionnaires were entered directly into the database by the researcher immediately after collection; the agreements and the questionnaire were kept in the researcher’s private office in a locked cabinet that could only be accessed by the researcher.

## 3. Results

### 3.1. General Characteristics of the Participants

The study included 139 men (56.0%) and 109 women (44.0%), with a mean age of 61.55 years. There were 109 (44.0%) high school graduates and 231 participants (93.1%) were married. There were 194 patients (78.2%) who underwent surgical treatment. There were 137 patients (55.2%) who experienced metastasis from the primary site, and 129 subjects (52.0%) received chemotherapy. The duration after diagnosis of pancreatic cancer was 2.26 years (Table 1).

### 3.2. Descriptive Statistics of the Main Variables

Among the measured variables included in this study, the skewness and kurtosis did not deviate from the assumption of normal distribution. All of the variables in this study maintained the univariate normality and thus met the conditions of normal distribution required to apply the structural equation model (Table 2).

### 3.3. Verification of the Model

To minimize the error when correlating a single measurement variable with a theoretical variable, the variable was calculated using the formula (1 − reliability) × variance of observation variable. The fit indices of hypothetical model for CFI, PGFI, and PNFI reached the recommended level, whereas χ^2^, χ^2^/DF, GFI, AGFI, and RMSEA did not reach the appropriate model level (Table 3).

#### 3.3.1. Modification of the Hypothetical Model

The model was modified by fitting it to the data through theoretical assessment and by modifying it to increase coincidence and simplicity. We used the CR and modified index considering the theoretical backgrounds and logical validity. In the hypothetical model, one pathway (social support → duration after diagnosis) in which the CR value was not significant was deleted. The error term variables age and social support bias, and the error term variables symptoms and perceived health status, which had a modification index of ≥5, were linked to curves and treated as unknown quantities under the assumption that covariance existed based on a logical basis. This resulted in the construction of a modified model that was simpler and clearer than the hypothetical model, and in which the fit measure was improved.

#### 3.3.2. Fidelity Verification of the Modified Model 

The fit indices of the modified model were χ^2^ = 249.72 (*p* < 0.001), df = 155, χ^2^/df = 1.61, GFI = 0.91, AGFI = 0.88, CFI = 0.98, RMSEA = 0.05, PGF = 0.67, and PNFI = 0.76, indicating that all fit indices, except for AGFI, met the standard level. This was significantly more accurate than the fit index of the hypothetical model. Therefore, this model was determined to be a suitable model that fit the actual data (Table 3). 

#### 3.3.3. Parameter Estimation of the Modified Model

**Figure 1:**
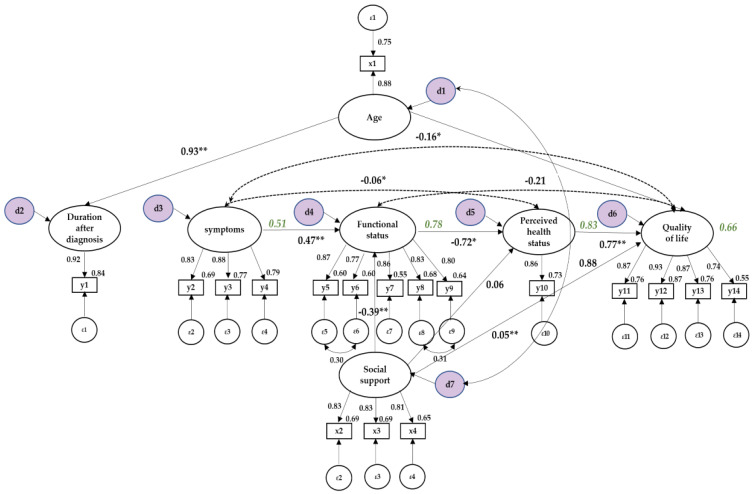
Path diagram for the modified model. * *p* < 0.05; ** *p* < 0.01; x1 = Age; x2 = Families; x3 = Friends; x4 = Important others; y1 = Duration after diagnosis; y2 = Physical symptoms; y3 = Depression; y4 = Anxiety; y5 = Physical functioning; y6 = Role functioning; y7 = Emotional functioning; y8 = Cognitive functioning; y9 = Social functioning; y10 = Perceived health status; y11 = Health-functional; y12 = Socioeconomic; y13 = Psychological-spiritual; y14 = Family.

Parameter estimation of the modified model showed that duration after diagnosis was influenced by age (β = 0.93, *p* = 0.002); the degree to which the age was explained by duration after diagnosis was 86%. Functional status was affected by social support; social support was positively correlated with functional status (β = −0.39, *p* = 0.003); symptoms were positively correlated with functional status (β = 0.47, *p* = 0.002); the degree to which functional status was explained by social support and symptoms was 78%. The perceived health status was correlated with social support (β = 0.06, *p* = 0.044); functional status was positively correlated with perceived health status (β = −0.72, *p* = 0.009); the degree to which perceived health status was explained by social support and functional status was 83%. The QoL was positively correlated with perceived health status (β = 0.77, *p* = 0.001); the degree to which the QoL was explained by the perceived health status was 66% (Figure 1).

### 3.4. Analysis of the Modified Model

The results of the analysis of the direct, indirect, and total effects of the exogenous variables of the modified model on the endogenous variables. The direct effects refer to the direct effects of exogenous variables on endogenous variables; the indirect effect refers to the effect of an exogenous variable that passes through one or more other variables, affecting the resulting variable; the total effect represents the sum of the direct and indirect effects. Among the 18 pathways of the model, seven pathways had significant direct effects, and 10 had significant total effects comprising direct and indirect effects. The age had direct effects on duration after diagnosis. Regarding functional status, social support and symptoms had direct effects. Regarding perceived health status, social support had direct and indirect effects, symptoms had indirect effects, and functional status had direct effects. For the QoL, the perceived health status had direct effects, and the age, social support, symptoms, and functional status had indirect effects (Table 4).

## 4. Discussion

In this study, we used the HRQoL model and a previous study by Ferrans et al. [3] to construct a structural model to explain the factors affecting the QoL of patients with pancreatic cancer and the causal relationships between these factors. The model showed that the perceived health status had direct effects on the QoL of patients with pancreatic cancer, and personal property, symptoms, functional status, and social support showed indirect effects. 

These variables explained approximately 63% of the QoL of patients with pancreatic cancer. In previous studies, self-care behaviors, depression, and perceived health status had an explanation power of 67.9% for the QoL of patients with stomach cancer; depression, self-esteem, fatigue, family support, uncertainty, self-efficacy, and subjective health status explained 67% of the QoL of patients with breast cancer and fatigue, pain, anxiety, social support, and anticancer treatment explained 66% of the QoL of patients with breast cancer [19]. Although the explanatory power of the present model was lower than that of previous studies, the fit of the model met the recommended level and was therefore appropriate for predicting the QoL of patients with pancreatic cancer. The differences between study results could be attributed to differences in type of cancer, severity of disease, treatment and recovery processes, measurement variables, and measurement tools.

In this model, the QoL score was 18.8, which was consistent with the results of Gupta, Lis, & Grutsch [20], who assessed the QoL of pancreatic cancer patients using the same measurement tools. However, in another study by Lis, Gupta & Grutsch [21] that measured the QoL of patients with prostate cancer, the overall QoL score was 22.8 points. In a study by Gupta et al. [22] that examined the QoL of colorectal cancer patients, the QoL score was 19.8 points. These results indicate that the QoL of pancreatic cancer patients is lower than that of patients with other cancers. The results of this study support those of previous studies showing that the QoL of pancreatic cancer patients is significantly lower than that of other cancer patients [23]. This highlights the importance of studies aimed at improving the QoL of patients with pancreatic cancer.

In the analysis of the effects of different variables on QoL, the health-functional domain had the lowest score, and the family domain had the highest score. This was consistent with the results reported by Lis, Gupta & Grutsch [21], which showed the lowest score in the health-functional domain and the highest score in the family domain. These results suggest that the QoL of pancreatic cancer patients was low in terms of health status, health care, energy for daily life, and the ability to care for themselves without help, whereas the QoL was high regarding the perception related to help from family members. The results of this study suggest that despite the problems faced by patients with pancreatic cancer during the treatment process, the support of family leads to positive perceptions regarding life.

In this study, the perceived health status of pancreatic cancer patients was the only factor with a direct effect on the QoL. In general, the perceived health status of cancer patients affects treatment outcomes and prognosis, and a greater awareness of one’s health results in a more positive QoL [6]. In this context, the results of this study were consistent with those of previous studies showing that the perceived health status of patients is an important variable with a positive effect on QoL. Ferrans et al. [3] also argued that perceived health status is an important conceptual variable that affects QoL, and stated that it is the combined result of all aspects related to QoL for the HRQoL. Because self-perception affects behavior, improving the health perception of individuals is important. This suggests that it would be useful to assess the perceived health status at the time of diagnosis; to implement this during the treatment process, one possible strategy would be to assess and modify the factors that affect the perceived health status.

In the present explanatory model, social support had a direct effect on the perceived health status, whereas the symptoms had an indirect effect. Social support directly affected the functional status and perceived health status, whereas it indirectly influenced the QoL. This suggests that although social support does not directly affect QoL, it influences QoL by functioning as a mediating factor that modulates the effects of functional status and perceived health status. These results are consistent with those of previous studies in which the social support perceived by cancer patients is positively correlated with the level of meaning or QoL [24]. Patients receiving high levels of social support have social skills to some extent, and higher levels of support are associated with fewer emotional symptoms, such as depression and anxiety, and decreased physical symptoms. In addition, social support is associated with improved health-seeking behaviors and higher QoL. 

In this study, family support scored higher than the support of friends and others in the sub-questions addressing social support. One explanation for this result is that the support of the family remains active when the survival rate decreases and the prognosis is unfavorable considering the nature of the disease. Accordingly, the importance of the support of family compared with that of other support systems underscores the need to actively seek the participation of the family in nursing interventions. Counseling and coaching programs for cancer patients under treatment and their families would improve the understanding of and adaptation to the treatment through actively sharing the process and mediating the communication between patients and their families, as well as helping to find solutions. The finding that the support of others was perceived as less valuable than the support of family and friends represents a challenge regarding the role of the nurse as a patient advocate or supporter. Thus, nurse practitioners involved in cancer education need to actively form a therapeutic relationship to provide individual counseling regarding the treatment process. 

Symptoms were identified as a factor that indirectly affects the perceived health status and directly affects the functional status. Although symptoms are not a factor that directly affects QoL, it acts as a mediating factor that modulates the effectiveness of the functional status and the perceived health status, which affects the QoL. These results were consistent with those of previous studies in which the physical and psychological symptoms experienced by pancreatic cancer patients were identified as the main factors affecting QoL. The symptoms and QoL of pancreatic cancer patients showed a statistically significant negative correlation, which is consistent with previous results showing that a greater experience with symptoms was correlated with a weaker functional status and a more negative perception of health and QoL [23]. This suggests that managing symptoms is important for improving the patient’s functional status and QoL. In addition, assessing and modulating the physical and psychological symptoms of patients is necessary during the disease and treatment processes. Emotional symptoms can be modulated by providing information regarding proper pain management, fatigue management, and a healthy lifestyle, and developing certain programs such as health recovery meditation, horticulture therapy, laughter therapy, and art therapy is also important during palliative care. 

The functional status acted as an indirect factor affecting the QoL; at the same time, it directly affected the perceived health status, and it was directly influenced by social support and symptoms. The functional status of cancer patients is related to prognosis, depression, and decision-making regarding treatment, and if the patient’s performance of a task declines, normal life becomes difficult, ultimately reducing QoL [24,25]. To improve the patient’s functional status and QoL, early intervention starting at the early stage of diagnosis is necessary by paying attention to the social support system and symptoms, which are the factors that directly affect the functional status. In particular, cancer patients are easily daunted by a poor disease prognosis and face problems related to various functional changes after returning home or to work. In this situation, the social support system could improve the functional status by providing physical and positive attitudes and health care. In addition, improving self-care using coaching techniques will contribute to improving the functional status by reducing the complications and side effects of treatment, as well as by improving the understanding of the disease.

This study is the first to establish a QoL model targeting pancreatic cancer patients in Korea. We identified multiple causal relationships among the various variables, which leads to an improved understanding of the role of nursing and contributes to improving nursing knowledge. The model verified in this study could provide the theoretical basis for future studies analyzing the factors related to QoL in other cancer patients. 

## 5. Conclusions

In this study, we identified the factors and pathways that affect the QoL of patients with pancreatic cancer to provide basic data for nursing intervention strategies aimed at improving the QoL of this patient population. For this purpose, we constructed a ‘QoL of patients with pancreatic cancer’ explanatory model based on the relationship between the identified factors.

To improve the perceived health status of patients with pancreatic cancer, it is necessary to assess the social support system, functional status, and symptoms from the time of diagnosis. The application of social support intervention programs to relieve specific symptoms and improve the functional status according to the conditions of patients may contribute to improving the QoL of pancreatic cancer patients. 

## 6. Limitations

The model constructed in this study was based on data obtained after convenience sampling aimed at pancreatic cancer patients in local hospitals, and the results need to be interpreted with caution. This study does not consider the characteristics of subjects according to the stage of pancreatic cancer, so there is a limitation in generalizing the study results. Therefore, to improve the QoL of patients with pancreatic cancer, a longitudinal study based on the timing of disease is needed to establish and control the variables affecting the treatment process.

Meanwhile, QoL comparisons are limited because of the lack of quantitative studies using valid and reliable tools to measure the QoL of patients with pancreatic cancer. Therefore, it is necessary to develop a tool to assess the disease-specific characteristics of pancreatic cancer patients. 

## Figures and Tables

**Table 1:** General characteristics of the participants (N = 248).

Characteristics	Categories	n (%)	Mean ± SD
Gender	Male	139 (56.0)	
	Female	109 (44.0)	
Age (years)			61.55 (9.85)
Education level	≤Middle school	52 (33.0)	
	High school	109 (44.0)	
	≥college	57 (23.0)	
Marital status	Married	231 (93.1)	
	Single	13 (5.2)	
	Others	4 (1.6)	
Primary Caregiver	Spouse	203 (81.9)	
	Children	34 (13.7)	
	Others	11 (4.4)	
Surgery	Yes	194 (78.2)	
	No	54 (21.8)	
Metastasis	Yes	137 (55.2)	
	No	111 (44.8)	
Chemotherapy	Yes	129 (52.0)	
	No	119 (48.0)	
Duration after diagnosis (yr)			2.26 (2.88)

**Table 2:** Descriptive statistics of the main variables (N = 248).

Characteristics	Categories	Mean ± SD	Range	Skewness	Kurtosis
Social support	Family	17.64 ± 1.94	14–20	−0.22	−1.15
	Friend	17.35 ± 2.07	13–20	−0.65	−0.58
	Others	15.74 ± 2.36	12–20	−0.02	−0.83
Symptoms	Depression	16.06 ± 1.66	12–19	−0.32	0.75
	Anxiety	16.77 ± 1.25	15–20	0.93	0.55
	Physical symptoms	42.04 ± 8.64	41.14–63.00	0.44	−0.53
Functional Status	Physical functioning	53.05 ± 9.82	39.67–66.23	−0.24	−1.55
	Role functioning	43.08 ± 10.80	33.00–64.13	1.39	0.41
	Emotional functioning	41.23 ± 10.35	23.33–65.37	0.98	0.11
	Cognitive functioning	59.45 ± 6.55	49.67–72.10	0.91	−0.31
	Social functioning	60.23 ± 9.84	33.33–71.51	−0.80	0.59
QoL	Health functional	15.01 ± 4.32	8.00–21.81	−0.15	−1.71
	Socioeconomic	19.18 ± 1.78	17.06–23.31	1.09	−0.15
	Psychological-spiritual	18.70 ± 2.29	16.50–23.21	0.65	−1.12
	Family	22.80 ± 2.89	18.70–28.50	0.01	−1.17

**Table 3:** Model fitness index for hypothetical and modified model (N = 248).

Goodness	χ^2^	DF	χ^2^/DF	*p*	GFI	AGFI	CFI	RMSEA	PGFI	PNFI
Evaluation criteria	–	–	<2	>0.05	≥0.90	≥0.90	≥0.90	≤0.05	≥0.50	≥0.50
Hypothetical model	459.14	156	2.94	<0.001	0.86	0.82	0.92	0.09	0.64	0.73
Modified model	249.72	155	1.61	0.064	0.91	0.88	0.98	0.05	0.67	0.76

GFI = Goodness of fit index; AGFI = Adjusted goodness of fit index; CFI = Comparative fit index; RMSEA = Root mean squared error of approximation; PGFI = Parsimonious goodness of fit index; PNFI = Parsimonious normed fit index.

**Table 4:** Direct Effect, Indirect Effect, and Total Effect in Modified Path Model (N = 248).

Variables	Categories	Modified Model			Direct Effect (*p*)	Indirect Effect (*p*)	Total Effect (*p*)
		SRW (SE)	T (*p*)	SMC			
Duration after diagnosis	Age	0.93 (0.06)	15.98 (<0.001) ***	0.86	0.93 (0.002) **		0.93 (0.002) **
Symptoms	Age	0.72(0.39)	1.75 (0.181)	0.51	0.73 (0.195)	−0.83 (0.761)	−0.10 (0.108)
	Social support	−0.10 (0.19)	−0.56 (0.573)		−0.10 (0.754)		−0.10 (0.754)
	Duration after diagnosis	−0.11 (0.27)	−0.37 (0.743)		−0.11 (0.829)		−0.11 (0.829)
Functional status	Age	0.11 (0.11)	0.78 (0.433)	0.78	0.11 (0.621)	0.17 (0.342)	0.28 (0.242)
	Social support	−0.39 (0.12)	−3.01 (0.003) **		−0.39 (0.003) **	0.70 (0.424)	−0.31 (0.003) **
	Symptoms	0.47 (0.06)	6.43 (<0.001) ***		0.47 (0.002) **		0.47 (0.002) **
Perceived health status	Age	0.17 (0.11)	1.04 (0.296)	0.83	0.17 (0.856)	0.24 (0.236)	0.41 (0.197)
	Social support	0.06 (0.12)	0.35 (0.028) *		0.06 (0.044) *	0.19 (0.017) *	0.25 (0.012) *
	Duration after diagnosis					0.88 (0.768)	0.88 (0.768)
	Symptoms					−0.06 (0.016) *	−0.06 (0.016) *
	Functional status	−0.74 (0.009)	−6.16 (<0.001) ***		−0.72 (0.009) *		−0.72 (0.022) *
Quality of life	Age	−0.21 (0.22)	−1.07 (0.283)	0.66	−0.21 (0.292)	0.20 (0.019) *	−0.14 (0.577)
	Social support	0.05 (0.23)	0.27 (0.785)		0.05 (0.839)	0.31 (0.005) **	0.36 (0.007) **
	Duration after diagnosis					0.88 (0.768)	0.88 (0.768)
	Symptoms					−0.16 (0.016) *	−0.16 (0.016) *
	Functional status					−0.01 (0.026) *	−0.01 (0.026) *
	Perceived health status	0.77 (0.28)	4.2 (<0.001) ***		0.77 (0.001) **		0.77 (0.001) **

* *p* < 0.05; ** *p* < 0.01; *** *p* < 0.001; SRW = Standardized regression weight; SMC = Squared multiple correlation.

## Data Availability

The data presented in this study are available on request from the corresponding author. The data are not publicly available due to restrictions e.g., privacy or ethical.
